# From Bad to Worse: Safety Behaviors Exacerbate Eating Disorder Fears

**DOI:** 10.3390/bs13070574

**Published:** 2023-07-11

**Authors:** Michelle Spix, Hanna Melles, Anita Jansen

**Affiliations:** Department of Clinical Psychological Science, Maastricht University, 6229 ER Maastricht, The Netherlands; h.melles@maastrichtuniversity.nl (H.M.); a.jansen@maastrichtuniversity.nl (A.J.)

**Keywords:** eating disorder, anorexia nervosa, bulimia nervosa, fear of weight gain, safety behaviors, behavior as information, vignette study

## Abstract

When evaluating ambiguous situations, humans sometimes use their behavior as a source of information (behavior-as-information effect) and interpret safety behaviors as evidence for danger. Accordingly, we hypothesized that eating disorder safety behaviors (restrictive eating, body checking, etc.) might aggravate fear and anxiety in individuals with an eating disorder. The present study tested to what extent eating disorder safety behaviors increase threat perception in individuals with and without an eating disorder. For this, 108 individuals with a self-reported eating disorder diagnosis and 82 healthy controls rated the dangerousness of several short situations. The situations systematically varied in the presence of eating disorder safety behaviors and danger information. As expected, all participants perceived situations in which the protagonist executed an eating disorder safety behavior as more threatening than situations without a safety behavior. This ‘behavior-as-information’ effect was equally strong in individuals with and without an eating disorder. Additionally, safety behaviors strengthened threat perception more in safe situations than in dangerous situations. To conclude, the presence of eating disorder safety behavior can increase threat perception regardless of whether individuals have an eating disorder or not. This makes eating disorder safety behaviors a potential risk factor for the development and maintenance of eating disorder fears.

## 1. Introduction

Fear and anxiety are core symptoms of eating disorders, including anorexia nervosa and bulimia nervosa [1,2,3]. Patients fear a variety of situations and stimuli including food, eating and (uncontrollable) weight gain, as well as its personal, physical and social consequences [4,5,6,7,8]. Despite the impact of fear and anxiety on the course and severity of eating disorders [9,10,11,12], as well as patients’ prognosis and their treatment outcomes [13,14], little is known about the mechanisms responsible for the development and maintenance of eating disorder fears.

While fear is an adaptive response when confronted with an imminent or objective danger, it becomes dysfunctional when triggered in safe situations and when causing harmful or disabling safety behaviors—referring to all types of actions intended to detect, avoid, escape or neutralize a feared outcome [15,16,17,18]. Individuals with an eating disorder engage in a wide array of safety behaviors to avoid (uncontrollable) weight gain: they refuse to eat high-calorie foods, reduce their calorie intake, skip meals, and narrow their food choices to a few ‘safe’ foods. Moreover, individuals with an eating disorder often neutralize the effect of calorie intake on body weight with excessive sporting, self-induced vomiting and taking laxatives. Additionally, they might check their body (e.g., measuring certain body parts), frequently weigh themselves or ritualize eating habits to reduce weight-related anxiety (for a detailed discussion of eating disorder safety behaviors see [3,8,19,20,21]). While eating disorder safety behaviors might help individuals to deal with their fear in the short run, they entail detrimental psychological and physiological long-term consequences [3].

Safety behaviors are not only a consequence of fear but might actively contribute to the development and persistence of fear and anxiety [22]. In a previous study, healthy participants who increased their use of appearance-related safety behaviors (e.g., checking appearance in the mirror, applying make-up and wearing camouflaging clothes) over the course of a week, became more dissatisfied with their bodies and interpreted ambiguous appearance-related situations as more threatening compared to a control group that was instructed to merely monitor their behavior over a similar time period [23]. In a similar vein, instructing healthy participants to perform safety behaviors related to contamination fear (e.g., disinfecting hands and surfaces) and health anxiety (e.g., checking body functions) intensified corresponding symptoms [17,24,25]. Additionally, laboratory studies showed that participants who thought they could reduce the intensity and duration of a painful heat stimulus with a button press (even though this safety behavior was ineffective) showed an increase in their pain-related fear and threat appraisals [26,27]. In sum, safety behaviors might play an important role in the development and maintenance of dysfunctional fear, anxiety, and threat appraisals.

A potential explanation for the bi-directional relationship between safety behaviors and fear is offered by the so-called ‘behavior-as-information’ effect [22,28,29,30]: individuals supposedly rely on their behavior as a source of information when drawing conclusions about the dangerousness of an (ambiguous) situation. In other words, they make inferences along the lines of, ‘If I avoid, there must be danger’ [28,29,30]. Drawing invalid conclusions about the dangerousness of a situation can hinder the correction of false threat beliefs, escalate fears and promote the generalization of fears to safe situations [3,31,32]. Thus, the tendency to rely on behavioral information when judging the dangerousness of a situation could constitute a vulnerability factor for the development of anxiety-related disorders. In line with this notion, studies by Gangemi et al., (2012) and van den Hout et al. (2014) found a greater ‘behavior-as-information’ effect in patients with anxiety disorders (obsessive compulsive disorder, panic disorder and social anxiety disorder) compared with healthy controls. In these studies, participants were asked to read different situations and to imagine themselves as the protagonist of these stories. Importantly, one half of the situations included an anxiety-specific safety behavior, while the other half did not. After reading each situation, participants indicated how dangerous they found the described situation. Patients perceived situations with safety behaviors as significantly more dangerous than controls, especially when more objective danger information was lacking [28,29].

Considering the frequent comorbidity [33,34], shared genetic vulnerabilities [35,36], and overlapping core symptoms between anxiety and eating disorders [10,37,38], patients with an eating disorder might also strongly rely on their safety behaviors when appraising the dangerousness of an (ambiguous) situation. When restricting food intake, patients might make inferences such as, ‘If I avoid high calorie foods, eating high calorie foods must be bad for me’. Consequently, they perceive eating high-calorie foods as dangerous and in turn become ever-more restrictive in their eating behaviors—a vicious cycle in which eating disorder fears and safety behaviors mutually reinforce each other. Thus, the ‘behavior-as-information’ effect could also constitute a vulnerability factor for the development, maintenance and deterioration of eating disorders. However, empirical research investigating the ‘behavior-as-information’ effect in the context of eating disorders is largely missing.

To address this gap in the literature, the present study investigates: (a) whether eating disorder-related safety behaviors influence how dangerous individuals perceive a situation and (b) whether individuals with an eating disorder show a heightened tendency to use eating disorder-related safety behaviors as a source of information compared with healthy controls. Our study largely followed the experimental design described in Gangemi et al. (2012) and van den Hout et al. (2014): individuals with an eating disorder and healthy controls read several short situations that systematically varied in the presence of eating disorder-related safety behaviors and objective danger information. After reading each situation, participants indicated how dangerous and pleasant they perceived the situation to be and if they wanted to experience such an event. We included the last two questions because many situations feared by individuals with an eating disorder (e.g., eating high-calorie foods) are appetitive for healthy individuals [39], so that only asking about danger might fail to fully capture the ‘behavior-as-information’ effect. Thus, participants’ ratings not only provide insights into their threat perception but also their appetitive responses towards the situation. We hypothesized that all participants evaluate situations with eating disorder safety behaviors as more dangerous and less pleasant than situations without safety behaviors (Hypothesis 1). Safety behaviors should increase threat perception and reduce appetitive responses to a greater extent in individuals with an eating disorder compared with healthy controls (Hypothesis 2). Lastly, we expected that the presence of objective danger information reduces the impact of safety behaviors on participants’ evaluation of the situations (Hypothesis 3).

## 2. Methods

### 2.1. Study Design

In the present quasi-experiment, individuals with and without an eating disorder rated several short situations in terms of danger, threat, pleasantness and their desire to experience the situation. We used a 2 × 2 × 2 mixed design, with participants’ diagnosis (eating disorder diagnosis vs. no diagnosis) as the between-subjects factor, and the description of safety behaviors (safety behavior vs. no safety behavior) and objective danger (dangerous vs. safe) in the situation as within-subjects factors. This resulted in four different versions per situation (see Table 1). Participants’ ratings of the situations were used as the main outcome measures. Additionally, we assessed several control variables: to verify participants’ diagnoses, we obtained information about their eating disorder symptoms; to account for the influence of other mental disorders on participants’ ratings, we assessed symptoms of depression and anxiety.

### 2.2. Participants

Individuals with an eating disorder and healthy controls participated in the study. Participants were recruited via social media and the research participation board of Maastricht University (SONA). Additionally, we invited patients who participated in some previous studies conducted by our research group to also participate in this study. To be included in the study participants needed to be female, at least 16 years old and speak Dutch or German. Healthy controls were excluded from the study if they reported to have undergone treatment for a mental disorder in the past three years. To be included in the patient group, participants had to indicate that they were diagnosed with an eating disorder by a professional practitioner and regularly engaged in eating disorder safety behaviors (i.e., restrictive eating, excessive sporting, or self-induced vomiting). Participants received course credits or a monetary reimbursement of € 7.50. Further information about the inclusion and exclusion of participants and the demographics of the final sample can be found in the Participants sub-section of the Results.

### 2.3. Materials

#### 2.3.1. Vignette Task

The structure of the vignette task was based on previous studies on the ‘behavior-as-information’ effect [28,29]. At the beginning of the vignette task, participants received the following instructions:

*“Below several events are described. We ask you to evaluate these events as if they are happening to you. There are no right or wrong answers; we are interested in the way you evaluate them. Try to identify with the events as much as possible and to imagine how you would feel. The descriptions sometimes resemble each other. Therefore, it is important that you read all descriptions very carefully from beginning to end.”*

Then, participants were presented with four situations, covering eating disorder symptoms related to eating, weight gain, body aversion and social judgement. We decided to include four situations to capture some variance in eating disorder fears (e.g., eating fatty foods in front of others, losing control when eating, and comments by others on weight gain) and safety behaviors (e.g., following eating rules, excessive exercising, body checking, skipping meals, and withdrawing from social contact). The situations varied in the presence of eating disorder-related objective danger (dangerous vs. safe) and in the presence of eating disorder-related safety behaviors (safety behavior vs. no safety behavior). Thus, participants read four versions of each situation (dangerous—safety behavior, safe—safety behavior, dangerous—no safety behavior, safe—no safety behavior). Situations were grouped into four blocks, so that different versions of the same situation never directly followed each other. The presentation order of the blocks was randomized across participants.

As an example, the situations on losing control while eating chocolate started as follows:

*“You come home after a long day. In the kitchen you see a large box of your favorite chocolate.”*

The dangerous—safety behavior situation continued as follows:

*“You eat a small piece of chocolate and leave the kitchen. After a few minutes, you find yourself again in the kitchen. You have an intense craving for chocolate. This time you get out the whole box and start to eat. You only stop once the box is empty. You put away the box, walk to the bathroom and make yourself throw up into the toilet.”*

The safe—safety behavior situation continued as follows:

*“You take the chocolate bars and eat two small pieces. You put away the box, walk to the bathroom and make yourself throw up into the toilet.”*

The dangerous—no safety behavior situation continued as follows:

*“You eat a small piece of chocolate and leave the kitchen. After a few minutes, you find yourself again in the kitchen. You have an intense craving for chocolate. This time you get out the whole box and start to eat. You only stop once the box is empty. You put away the box and sit down in front of the tv in the living room in order to watch your favorite tv show.”*

The safe—no safety behavior situation continued as follows:

*“You take the chocolate bars and eat two small pieces. You put away the box and sit down in front of the tv in the living room in order to watch your favorite tv show.”*

The other situations can be found in Supplementary Material S1.

After reading each situation, participants rated them on different aspects. The first question functioned as a manipulation check; here, participants rated on a Visual Analogue Scale (VAS) how well they could imagine themselves being in the situation. A filler item was included to prevent participants from remembering their previous ratings; here, participants indicated how they had imagined a specific detail of the situation (e.g., “If you imagine yourself in this situation, is your kitchen big or small?”). Then, participants rated on VASs how dangerous (0 = “Not dangerous at all”; 100 = “Very dangerous”), threatening (0 = “Not threatening at all”; 100 = “Very threatening”) and pleasant (0 = “Not pleasant at all”; 100 = “Very pleasant”) they perceived the situation to be. Additionally, they indicated how much they would like to experience such a situation (0 = “Not at all”; 100 = “Very much”).

#### 2.3.2. Questionnaires

**Eating Disorder Examination Questionnaire (EDE-Q).** The EDE-Q 6.0 consists of 28 items and assesses eating disorder psychopathology over the past 4 weeks [40]. It includes four subscales: restraint (e.g., “Have you been deliberately trying to limit the amount of food you eat to influence your shape or weight (whether or not you have succeeded)?”), shape concern (e.g., “How dissatisfied have you been with your shape?”), eating concern (e.g., “Have you had a definite fear of losing control over eating?”) and weight concern (e.g., “Has your weight influenced how you think about (judge) yourself as a person?”). Items are rated on a seven-point Likert scale, reflecting how often or how intense the individual experienced eating disorder symptoms in the past 28 days (0 = No days/Not at all; 6 = Every day/Markedly). Additionally, the EDE-Q includes six open questions about the frequency of binge eating and associated compensatory behaviors. Subscale scores are calculated by averaging the respective items; the global score is calculated by averaging across subscales. A higher score indicates more severe eating disorder symptomatology. In the present study, the subscales and global score of the EDE-Q showed good internal consistency with alpha > 0.86. We used the German [41] and Dutch (by the last author) translations of the EDE-Q.

**DSM-5 criteria checklist.** This questionnaire was developed by the last author to obtain more detailed information about eating disorder symptoms over the past week and the last three months (for the diagnosis check). The 73 items are derived from the DSM-5 criteria for anorexia nervosa, bulimia nervosa, and binge eating disorder [1] and focus on the frequency of eating disorder-related cognitions (e.g., “I am too fat”), feelings (e.g., “In the past week, I felt miserable after eating too much”) and behaviors (e.g., “In the past week, I deliberately vomited to compensate for what I had eaten”). Items are rated on a five-point Likert scale, reflecting the frequency of eating disorder symptoms over the past week or the last three months (“0 times (never)”/“(almost) never” to “7 times or more”/“(almost) always”). The DSM-5 criteria checklist is not intended for the calculation of summary statistics, such as subscale or global scores. Instead, we used participants’ answers on specific items—relevant for their self-reported diagnosis—to inform the diagnosis check. The questionnaire has been used in previous research from our laboratory to assist in verifying self-reported diagnoses [7]. The questionnaire is available upon request.

**Short Depression Anxiety Stress Scale (DASS-21).** The DASS-21 assesses the occurrence of negative emotional states over the past week [42]. It covers three subscales: the depression scale asks about dysphoria, hopelessness, devaluation of life, self-depreciation, and anhedonia; the anxiety scale covers autonomic arousal, skeletal muscle effects and subjective experiences of anxious affect; and the stress scale addresses difficulties relaxing, impatience and over-reactivity. The DASS-21 consists of 21 items, which are rated on a 4-point scale (0—“Did not apply to me at all”; 3—“Applied to me very much”). Subscale scores are calculated by summing the respective item scores and multiplying the result by two. A higher score indicates more severe symptoms of anxiety, depression, or stress. In the present study, the subscales scores of the DASS-21 showed good internal consistency with alpha > 0.88. We used the German [43] and Dutch [44] translation of the DASS-21.

### 2.4. Procedure

The entire study took place online and was presented with the programs SOTO and Qualtrics. After starting the study, participants could choose their preferred language from Dutch and German. Then, participants read the information letter and provided informed consent. Next, they answered questions on demographics (age and gender) and—if applicable—provided more detailed information on their eating disorder diagnosis and current treatment. Additionally, all participants had to indicate whether they had been in treatment for another mental disorder besides an eating disorder in the past three years. After these questions, participants completed the vignette task. Then, they filled in the EDE-Q, the DSM-5 criteria checklist and the DASS-21. Participants also reported their current height and weight, as well as their lowest weight since their 18th birthday. We included two check questions to detect inattentive answering and bot activity. At the end of the study, participants were thanked for their participation and received their reimbursement. Overall, the study took between 30 and 45 min. The study was approved by the Ethical Committee of the Faculty of Psychology and Neuroscience at Maastricht University.

### 2.5. Statistical Analysis Plan

Before starting the statistical analyses, the authors (H.M. and M.S.) checked whether participants’ self-reported eating disorder diagnoses fitted the clinical information provided in the EDE-Q and the DSM-5 criteria checklist. After checking the first cases together, the researchers completed the assessment of the remaining diagnoses separately. When comparing the outcomes, the authors agreed on 85.6% of the cases (101 out of 118 participants). The remaining unclear cases were discussed and resolved together. In cases where participants’ self-reported diagnosis did not align with their clinical information, diagnoses were adjusted.

All statistical analyses were conducted in R (version 4.1.2). To check whether the safety behaviors described in the vignettes really represented behavior relevant to individuals with an eating disorder, we ran a linear mixed model analysis (LMM) with imagination ratings as the dependent variables. Participants’ diagnostic status (patients vs. healthy controls), the presence of safety behaviors in the situation (SB vs. no SB) and their interaction served as fixed effects. We used LMMs for the analysis of the present data as they are a powerful and flexible tool for analyzing repeated measures data [45,46] and have been successfully employed in other studies on fear and avoidance in eating disorders [47,48].

Then, we calculated bivariate correlations between ‘danger’, ‘threat’, ‘pleasantness’ and ‘want for experience’ ratings. Based on the high correlations (see Table 2), we combined ‘danger’ and ‘threat’ ratings into an average score (from now on called ‘threat’ ratings). We tested our hypotheses by running several LMMs with ‘threat’, ‘pleasantness’ and ‘want for experience’ ratings as dependent variables. To see how safety behaviors affected ratings in participants with and without an eating disorder, we included their diagnostic status, the presence of safety behaviors in the situation, and their interaction term as fixed effects in the model. Additionally, we tested whether the ‘behavior-as-information’ effect differed between situations with and without objective danger information. Therefore, we added the presence of danger (dangerous vs. safe) to the LMMs. To test whether comorbid anxiety or depression influenced the results, we reran the previously described analyses with the DASS-21 anxiety and depression subscales as covariates. To further check the robustness of our results, we repeated the analyses after excluding: (a) patients with other specified feeding or eating disorders (OSFED) and (b) healthy controls with an EDEQ global score above 2.

In all models, we included random effects per participant; we included random slopes when they improved model fit, as indicated by a significantly lower log likelihood. The LMMs were computed using the *lme4* package [49]. To follow up on significant findings, we calculated marginal means using the *emmeans* package [50] and adjusted for multiple comparisons with the Tukey method. Cohen’s d was calculated using the *effect size* package [51]. We considered findings with a *p*-value lower than 0.05 as significant. The hypotheses and analyses were pre-registered under https://aspredicted.org/7rz3m.pdf.

## 3. Results

### 3.1. Participants

**Figure 1:**
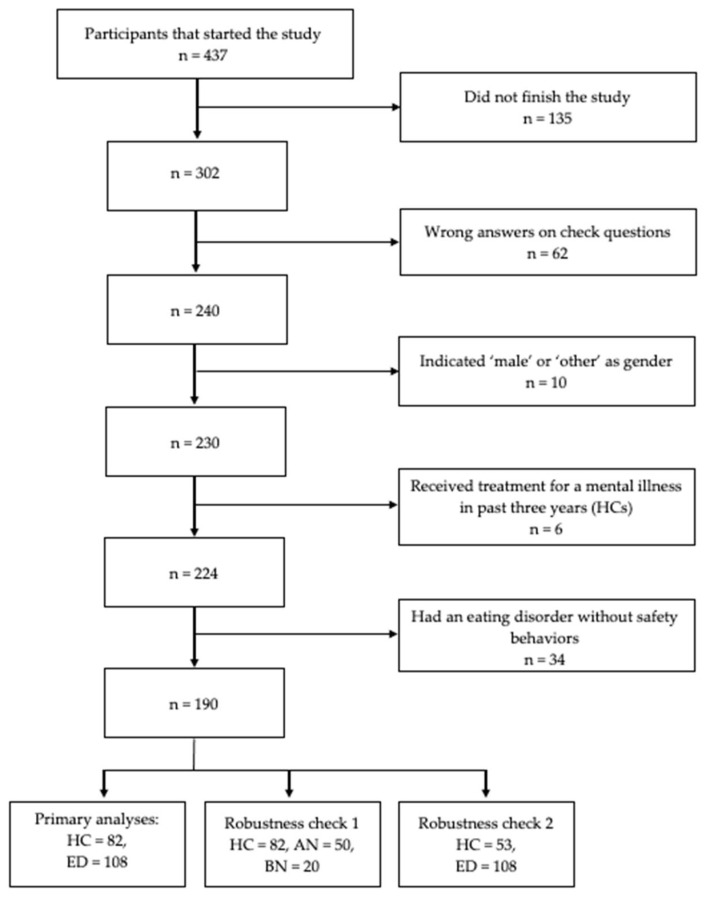
Decision tree for the exclusion of participants from the analysis. *Note.* HC = healthy control; ED = individuals with an eating disorder; AN = anorexia nervosa; BN = bulimia nervosa.

The online study was started by 437 individuals. We excluded 213 participants as they did not finish the study (n = 135), provided wrong answers on the check questions (n = 62) or did not meet the inclusion criteria (n = 16). After looking at the clinical information of the remaining 142 participants who had indicated an eating disorder, we excluded 34 individuals that did not report any compensatory safety behaviors in the EDE-Q and DSM-5 symptom checklist. We provide an overview of our reasons for excluding participants in Figure 1. The final sample consisted of 82 healthy controls and 108 patients with an eating disorder (50 patients with anorexia nervosa, 20 patients with bulimia nervosa, 38 patients with OSFED). For additional information on sample characteristics, see Table 3.

### 3.2. Manipulation Check

Individuals with an eating disorder could imagine themselves equally well in situations with (M = 73.07, SD = 30.24) and without safety behaviors (M = 70.72, SD = 28.71); diagnostic status x safety behavior interaction (*F*_(1, 187.86)_ = 187.716, *p* < 0.001). Healthy controls were less able to imagine themselves in situations with safety behaviors (M = 39.41, SD = 34.40) compared with situations without safety behaviors (M = 74.97, SD = 23.91); safety behavior (*F*_(1, 187.77)_ = 290.247, *p* < 0.001). Post-hoc tests confirmed that patients could better imagine the situations with safety behaviors than healthy controls (*β* = −33.67, SE = 2.76, *t*_(188)_ = −12.22, *p* < 0.001, *d* = −1.78). Thus, patients perceived the safety behaviors included in the situations as more realistic than healthy controls.

### 3.3. How Do Safety Behaviors Affect Threat Perception in Individuals with an Eating Disorder and Healthy Controls? (Hypothesis 1 and 2)

Individuals with an eating disorder perceived the situations as more threatening than healthy controls; diagnostic status (*F*_(1, 188)_ = 194.51, *p* < 0.001). Individuals with an eating disorder and healthy controls perceived situations as more threatening when they included safety behaviors; safety behavior (*F*_(1, 2848)_ = 44.93, *p* < 0.001). The interaction effect was not significant (*p* = 0.079). Thus, the presence of safety behaviors affected participants’ threat perception of the situations (behavior-as-information effect) irrespective of their diagnosis. For (standardized) regression coefficients, consult Supplementary Material S2.

### 3.4. Do Safety Behaviors Affect Threat Perception Differently in Dangerous vs. Safe Situations? (Hypothesis 3)

**Figure 2:**
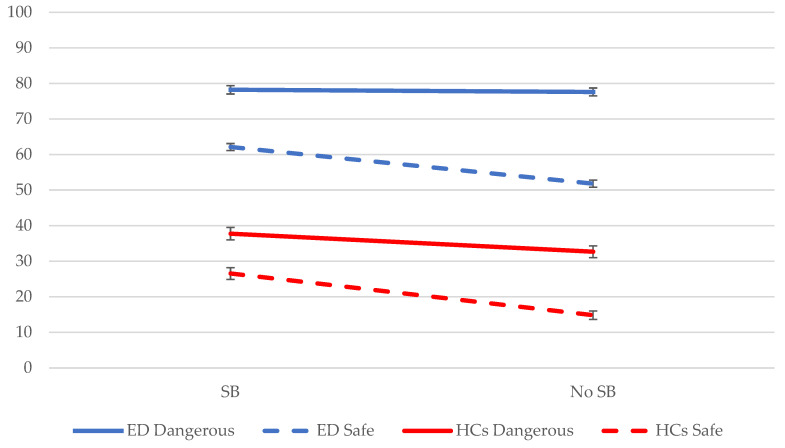
Mean threat ratings across the different variations of the situations for individuals with an eating disorder (ED) and healthy controls (HCs). *Note.* SB = safety behavior; blue solid line: eating disorder group and dangerous situation; blue dashed line: eating disorder group and safe situation; red solid line: healthy controls and dangerous situation; red dashed line: healthy controls and safe situation.

Participants perceived the ‘dangerous’ situations as more threatening than the ‘safe’ situations; presence of danger (*F*_(1, 188)_ = 84.02, *p* < 0.001). This effect was larger in individuals with an eating disorder than in healthy controls; diagnostic status x presence of danger (*F*_(1, 188)_ = 9.37, *p* = 0.003). Individuals with an eating disorder and healthy controls perceived the ‘safe’ situations with safety behaviors as more dangerous than ‘safe’ situations without safety behaviors; safety behavior (*F*_(1, 188)_ = 51.93, *p* < 0.001). The ‘behavior-as-information’ effect was smaller in ‘dangerous’ situations (see Figure 2); safety behavior x presence of danger (*F*_(1, 2468)_ = 9.43, *p* = 0.002). Post-hoc tests showed that patients and healthy controls displayed a ‘behavior-as-information’ effect in ‘safe’ situations (EDs: *β* = −10.32, SE = 1.39, *t*_(620)_ = −7.45, *p* < 0.001, *d* = −0.30; HCs: *β* = −11.72, SE = 1.59, *t*_(620)_ = −7.37, *p* < 0.001, *d* = −0.30), while only healthy controls presented it in ‘dangerous’ situations (EDs: *p* = 0.655, *d* = −0.02; HCs: *β* = −5.06, SE = 1.59, *t*_(620)_ = −3.18, *p* = 0.002, *d* = −0.13). In sum, the danger information included in the situations affected the strength of the ‘behavior-as-information’ effect: safety behaviors increased participants’ threat perception more strongly in safe situations compared with dangerous situations.

Overall, we found a similar pattern of results for ‘pleasantness’ and ‘want for experience’ ratings. Participants perceived situations with safety behaviors as less pleasant and reported a lower desire to be in this situation compared with situations that included no safety behaviors. Furthermore, this reduction in ratings was stronger in safe than in dangerous situations. Detailed results for ‘pleasantness’ and ‘want for experience’ ratings are presented in Supplementary Material S2.

### 3.5. Robustness Checks

The previously described pattern of results did not change after accounting for symptoms of anxiety and depression. Furthermore, excluding participants with OSFED and healthy controls with an EDEQ score above two (n = 29) from our sample did not change the outcomes of the LMM analyses. Thus, our findings proved robust across different specifications of the model and the sample.

For a detailed overview of the robustness checks, consult Supplementary Material S2.

## 4. Discussion

The present study investigated to what extent eating disorder safety behaviors would increase threat perception in individuals with and without an eating disorder. We found that individuals with an eating disorder perceived all situations as more threatening than healthy controls. Moreover, objectively dangerous situations were rated as more threatening than objectively safe situations by all participants, and this effect was more pronounced in individuals with an eating disorder. In line with our hypothesis, all participants rated situations in which the protagonist executed a safety behavior as more threatening than the same situations without safety behaviors, i.e., the ‘behavior-as-information’ effect. When we look at the objectively safe vs. dangerous situations, all participants rated the safe situations with safety behaviors as more threatening than the same safe situations without safety behaviors. The healthy controls also rated the objectively dangerous situations with safety behaviors as more threatening than the objectively dangerous situations without safety behaviors. However, contrary to what we expected, safety behaviors did not affect the threat perception of dangerous situations in participants with an eating disorder. This may be a ceiling effect: the dangerous situations were, overall, rated as highly threatening by the eating disorder group. A similar pattern of results was found when participants evaluated the situations on positive attributes: they perceived situations with safety behaviors as less pleasant and attractive than identical situations without safety behaviors.

Participants with an eating disorder rated all situations—regardless of safety behaviors and additional danger information—as more threatening than healthy controls. These findings are in line with previous studies: individuals with eating disorders perceive a wide range of disorder-related situations and stimuli as threatening (e.g., weight gain, loss of control, social judgement). Fears and anxiety appear to be highly prevalent across all eating disorder diagnoses and they are considered by some experts to be core symptoms of eating disorders [4,6,7,10,38]. By triggering safety behaviors—aimed at neutralizing or preventing feared situations and/or stimuli—the eating disorder fears can cause dangerous physical consequences, such as severe underweight, limit the range of patients’ daily activities, and reduce their quality of life [3,15,52].

Importantly, our findings may indicate that safety behaviors in eating disorders are not only a response to eating disorder fears but also play an active role in their onset and exacerbation. The presence of eating disorder safety behaviors in a situation increased participants’ perception of danger and threat, so it seems safety behaviors served as a source of information when making inferences about the situation (behavior-as-information effect) [22,28,29]. Contrary to our expectations, there was no difference in the strength of the ‘behavior-as-information’ effect between individuals with and without an eating disorder; all participants interpreted eating disorder safety behaviors as a sign of threat whether they were diagnosed with an eating disorder or not. Accordingly, anyone who uses safety behaviors to prevent weight gain would make inferences along the lines of, ‘If I avoid weight gain, weight gain must be dangerous for me.’ In turn, a stronger fear of weight gain should trigger more frequent and extreme safety behaviors—the beginning of a harmful vicious cycle. Thereby, engaging in eating disorder safety behaviors, such as restrictive eating and excessive sporting, presents a risk factor for the development and maintenance of eating disorders.

In line with our expectations and previous studies by Gangemi et al., (2012) and van den Hout et al., (2014), we found that safety behaviors increased participants’ threat ratings more strongly for objectively safe situations than for objectively dangerous situations. These findings suggest that the ‘behavior-as-information’ effect is more relevant under high ambiguity: when individuals are inconclusive about the danger of a situation, they incorporate safety behaviors as additional evidence in their judgements; when individuals perceive a situation as dangerous based on more objective information, the additional knowledge offered by safety behaviors is redundant. Another explanation for the present findings is offered by cognitive dissonance reduction theory [53,54]. While safety behaviors are adaptive when objective danger is present, engaging in safety behaviors in the absence of danger should create cognitive dissonance due to a large mismatch between individuals’ beliefs about the situation and their behavior. To reduce this aversive state, individuals align their beliefs and behavior, so that they perceive the originally safe situation as more threatening [30]. A stronger ‘behavior-as-information’ effect for safe or ambiguous situations could make the use of safety behaviors especially harmful when eating disorder fears are developing or remitting, for example at disorder onset or after treatment. To rule out that methodological artifacts such as ceiling effects underlie a smaller ‘behavior-as-information’ effect in objectively dangerous situations, future studies should use a less intense or more nuanced danger manipulation and thoroughly pilot dangerous situations, to ensure that they evoke a sufficient variance in ratings.

The present findings also have clinical implications. As eating disorder safety behaviors appear to worsen eating disorder fears, treatments aiming for fear reduction (e.g., exposure therapy) also need to identify and reduce patients’ safety behaviors. Studies showed that reducing fear does not suffice to eliminate safety behaviors, thus calling for new interventions that specifically target safety behaviors [55,56]. For example, therapists could encourage patients to approach feared situations by drawing attention to rewarding consequences [55,57]. Stimulating approach behaviors, i.e., exposure with response prevention, offers a potential route to use the ‘behavior-as-information’ effect in an adaptive way [30]: when confronting a feared situation, such as eating high-calorie foods, patients may learn to make more adaptive inferences such as, ‘If I eat high calorie foods, high calorie foods cannot be that dangerous.’ Reducing safety behaviors and fostering approach behaviors could therefore be important elements in the treatment of eating disorders. These behavioral interventions could be combined with cognitive techniques that help patients to modify unhelpful thoughts and to rely on more valid sources of information. More research is needed to identify the best combination of cognitive and behavioral treatment approaches to reduce the ‘behavior-as-information’ effect.

As eating disorder safety behaviors also aggravate threat perception in healthy individuals, eating disorder prevention programs might profit from directly targeting safety behaviors. Educating participants about the detrimental consequences of their safety behavior use and equipping them with tools for behavior change [58] might help to break the vicious cycle between safety behaviors and fear before it can gain momentum.

### Limitations and Future Research

The present study has some limitations. Firstly, though scores on the EDE-Q and DSM-5 criteria checklist indicate the presence of eating disorder psychopathology in the eating disorder group, participants’ eating disorder diagnoses were not obtained by a proper diagnostic interview but based on carefully collected and checked self-reports. Similarly, we checked but cannot guarantee that healthy controls were without mental issues at the time of testing. To reduce this risk, we repeated the analyses without the healthy controls who reported somewhat higher levels of eating disorder psychopathology, and we included symptoms of anxiety and depression as covariates in the analyses. Our findings remained remarkably stable across these checks, suggesting that the effects are quite robust. Secondly, since the present study only included women and focused on certain eating disorder safety behaviors, conclusions about the ‘behavior-as-information’ effect in men or patients with binge eating disorder are not warranted. Therefore, future research should include more diverse samples regarding gender, age, cultural background and eating disorder diagnosis to increase the generalizability and clinical utility of the present finings. Thirdly, the online setting of the study possibly affected the quality of participants’ answers. As we did not control where, when and how participants filled in the vignette task and the questionnaires, they might have completed the study in a noisy and distracting environment. To reduce the risk that inattentive reading and incorrect answers would affect the validity of our data, we followed current guidelines for online research and included several check questions [59]. At the same time, the online setting potentially encouraged participants to disclose more personal information and to provide less socially desirable answers due to a greater level of anonymity and the possibility of completing the study in a comfortable environment [60]. Future research might want to replicate the present study using a pen-and-paper format to rule out any undue influence of the online setting on the obtained results. Lastly, the vignette design of the study might limit its ecological validity: imagining a behavior is different from really performing it [61]. Accordingly, the effect of eating disorder safety behaviors on eating disorder fears could play out differently in everyday life. To better understand the bi-directional relationship between safety behaviors and fear, future studies could have healthy participants perform eating disorder-related safety behaviors in a controlled setting.

## 5. Conclusions

The present study shows that the imagined carrying out of safety behaviors increases threat perceptions in individuals with and without eating disorders and, thereby, supports the notion that eating disorder-related safety behaviors may play a role in the development and maintenance of eating disorder fears.

## Figures and Tables

**Table 1:** Overview of the different versions per situation based on the combinations of within-subject factors (description of safety behaviors × description of objective danger).

Factor		Description of Objective Danger	
		Dangerous	Safe
Description of safety behavior	Safety behavior	Safety behavior—Dangerous	Safety behavior—Safe
	No safety behavior	No safety behavior—Dangerous	No safety behavior—Safe

**Table 2:** Correlations between ‘danger’, ‘threat’, ‘pleasantness’ and ‘want for experience’ ratings.

Variable	1.	2.	3.
Danger			
Threat	0.86 *		
Pleasantness	−0.51 *	−0.55 *	
Want for experience	−0.41 *	−0.44 *	0.77 *

*Note.* * *p* < 0.001.

**Table 3:** Sample characteristics (mean and standard deviation) for individuals with an eating disorder and healthy controls.

	HC (n = 82)	ED (n = 108)	*t*	df	*p*
Age	25.11 (6.54)	28.16 (10.17)	2.51	183.40	0.013
BMI	23.13 (3.59)	19.20 (3.54)	−7.46	185	<0.001
Lowest BMI After 18th Birthday	20.71 (2.61)	15.90 (3.22)	−10.93	186	<0.001
Illness Duration (Years)	-	11.11 (8.86)	-	-	-
Currently in Treatment (%)	-	69 (63.9%)	-	-	-
Treatment Duration (years)	-	7.12 (6.48)	-	-	-
EDEQ Global Score	1.56 (1.34)	4.21 (1.13)	14.47	157.87	<0.001
EDEQ Eating Restraint	1.15 (1.26)	4.11 (1.37)	15.29	188	<0.001
EDEQ Eating Concerns	0.99 (1.14)	3.40 (1.37)	13.19	185.94	<0.001
EDEQ Weight Concerns	1.97 (1.68)	4.50 (1.33)	11.17	150.07	<0.001
EDEQ Shape Concerns	2.11 (1.65)	4.83 (1.14)	12.73	136.64	<0.001
DASS Depression	8.73 (9.35)	24.67 (11.54)	10.49	186.41	<0.001
DASS Anxiety	7.02 (7.73)	17.79 (10.90)	7.94	185.95	<0.001
DASS Stress	11.27 (9.80)	23.87 (9.79)	8.77	187	<0.001

*Note.* HC = healthy control; ED = individuals with an eating disorder; cut-off scores for ‘normal’ label on DASS Depression: 9, DASS Anxiety: 7 and DASS Stress: 14.

## Data Availability

The data presented in this study are available on request from the corresponding author. The data are not publicly available due to privacy reasons.

## Supplementary Materials

The following supporting information can be downloaded at: https://www.mdpi.com/article/10.3390/bs13070574/s1, S1 Vignettes; S2.1 Results for Pleasantness and Want for experience, S2.2 Parameter information for the mixed model analyses, S2.3 Robustness checks.
