# Supplementary material for: From Bad to Worse: Safety Behaviors Exacerbate Eating Disorder Fears
Source: Behav Sci (Basel). 2023 Jul 11;13(7):574. doi: 10.3390/bs13070574 (PMC10376478; doi:10.3390/bs13070574)
Supplement: Supplementary file 1 [file behavsci-13-00574-s001.zip › Supplementary material S1.pdf]

Supplementary material S1 - Vignettes

Instructions at the beginning of the task:

Below a number of events is described. We ask you to evaluate these events as if they are happening to you. There are no right or wrong answers; we are interested in the way you evaluate this. Try to identify with the events as much as possible and to imagine how you would feel.

The descriptions sometimes resemble each other. Therefore, it is important that you read all descriptions very carefully from beginning to end.

Situation 1 – Holidays with family:

<u>Version 1 (dangerous - safety behavior):</u>

This afternoon you came home from a three-week long holiday with your family.
Every evening of the past three weeks you and your family went out for dinner or ate at the hotel buffet. You always got something hearty and fatty for the main course and sometimes something sweet for dessert. The portions were also lot larger than what you normally eat at home and every evening you felt quite full after dinner.
After unpacking your bags, you put on your sports clothes and go for a long run. When you come home exhausted, you decide to go jogging every day from now on.

<u>Version 2 (dangerous - no safety behavior):</u>

This afternoon you came home from a three-week long holiday with your family.
Every evening of the past three weeks you and your family went out for dinner or ate at the hotel buffet. You always got something hearty and fatty for the main course and sometimes something sweet for dessert. The portions were also lot larger than what you normally eat at home and every evening you felt quite full after dinner. After unpacking your bags, you meet with some friends.

<u>Version 3 (safe – safety behavior):</u>

This afternoon you came home from a three-week long holiday with your family.
In the past weeks you often cooked for your family or prepared meals for yourself. It was even easier for you to follow your eating rules during the trip than at home. After unpacking your bags, you put on your sports clothes and go for a long run. When you come home exhausted, you decide to go jogging every day from now on.

<u>Version 4 (safe - no safety behavior):</u>

This afternoon you came home from a three-week long holiday with your family.

In the past three weeks you often cooked for your family or prepared meals for yourself. It was even easier for you to follow your eating rules during the trip than at home. After unpacking your bags, you meet with some friends.

Situation 2 – Eating chocolate:

Version 1 (dangerous - safety behavior):

You come home after a long day. In the kitchen you see a large box of your favorite chocolate.
You eat a small piece of chocolate and leave the kitchen. After a few minutes, you find yourself again in the kitchen. You have an intense craving for chocolate. This time you get out the whole box and start to eat. You only stop once the box is empty. You put away the box, walk to the bathroom and make yourself throw up into the toilet.

Version 2 (dangerous – no safety behavior):

You come home after a long day. In the kitchen you see a large box of your favorite chocolate.
You eat a small piece of chocolate and leave the kitchen. After a few minutes, you find yourself again in the kitchen. You have an intense craving for chocolate. This time you get out the whole box and start to eat. You only stop once the box is empty. You put away the box and sit down in front of the tv in the living room in order to watch your favourite tv show.

Version 3 (safe - safety behavior):

You come home after a long day. In the kitchen you see a large box of your favorite chocolate.
You take the chocolate bars and eat two small pieces. You put away the box, walk to the bathroom and make yourself throw up into the toilet.

Version 4 (safe – no safety behavior):

You come home after a long day. In the kitchen you see a large box of your favorite chocolate.
You take the chocolate bars and eat two small pieces. You put away the box and sit down in front of the tv in the living room in order to watch your favourite tv show.

Situation 3 – Dinner with friends:

Version 1 (dangerous - safety behavior):

You have been invited for dinner by one of your friends. After a short chat, all of you sit down at the dinner table. Your friend puts a large pot of cheese fondue, bread and vegetables on the table.
It looks like way too much food for the small group that you are with. After eating a few pieces of bread with cheese and vegetables you are already full. When you indicate that you have finished eating, your friends urge you to eat more as food should not be wasted. Once the cheese fondue is finally emptied, you feel bloated.
Soon after finishing dinner you excuse yourself and leave your friend's house. Once you are home again you get a tape measure and measure the circumference of your stomach and legs. The next day you skip breakfast and lunch. You decide to fast for the full day.

Version 2 (dangerous – no safety behavior):

You have been invited for dinner by one of your friends. After a short chat, all of you sit down at the dinner table. Your friend puts a large pot of cheese fondue, bread and vegetables on the table.
It looks like way too much food for the small group that you are with. After eating a few pieces of bread with cheese and vegetables you are already full. When you indicate that you have finished eating, your friends urge you to eat more as food should not be wasted. Once the cheese fondue is finally emptied, you feel bloated.
After clearing the table, you and your friends play a board game together.

Version 3 (safe - safety behavior):

You have been invited for dinner by one of your friends. After a short chat, all of you sit down at the dinner table. Your friend puts a large pot of cheese fondue, bread and vegetables on the table.
Over the course of the meal, you eat some vegetables and a piece of bread, that you dip into the cheese fondue.
Soon after finishing dinner you excuse yourself and leave your friend's house. Once you are home again you get a tape measure and measure the circumference of your stomach and legs. The next day you skip breakfast and lunch. You decide to fast for the full day.

Version 4 (safe – no safety behavior):

You have been invited for dinner by one of your friends. After a short chat, all of you sit down at the dinner table. Your friend puts a large pot of cheese fondue, bread and vegetables on the table.
Over the course of the meal, you eat some vegetables and a piece of bread, that you dip into the cheese fondue.
After clearing the table, you and your friends play a board game together.

Situation 4 – Meeting friend in park

Version 1 (dangerous - safety behavior):

On a free morning you go for a walk in the park. You run into an old friend that you haven't seen for a while.
After greeting each other, your friend looks at you and says: 'You look a bit different. You have gained some weight, haven't you? How much it, 3 or 4 kilos?'. After a bit of small talk, you make up an excuse to leave. You say goodbye to your friend and go straight home. You get your phone, and you cancel all appointments with friends and family in the upcoming days. You have decided to stay home for the rest of the week.

Version 2 (dangerous – no safety behavior):

On a free morning you go for a walk in the park. You run into an old friend that you haven't seen for a while.
After greeting each other, your friend looks at you and says: 'You look a bit different. You have gained some weight, haven't you? How much it, 3 or 4 kilos?' You continue to chat for a while. As you say goodbye, you and your friend make plans to see each other again next week.

Version 3 (safe - safety behavior):

On a free morning you go for a walk in the park. You run into an old friend that you haven't seen for a while.
After greeting each other, your friend looks at you and says: 'You look a lot healthier than the last time I saw you!' After a bit of small talk, you make up an excuse to leave. You say goodbye to your friend and go straight home. You get your phone, and you cancel all appointments with friends and family in the upcoming days. You have decided to stay home for the rest of the week.

Version 4 (safe – no safety behavior):

On a free morning you go for a walk in the park. You run into an old friend that you haven't seen for a while.
After greeting each other, your friend looks at you and says: 'You look a lot healthier than the last time I saw you!' You continue to chat for a while. As you say goodbye, you and your friend make plans to see each other again next week.
