# Supplementary material for: From Bad to Worse: Safety Behaviors Exacerbate Eating Disorder Fears
Source: Behav Sci (Basel). 2023 Jul 11;13(7):574. doi: 10.3390/bs13070574 (PMC10376478; doi:10.3390/bs13070574)
Supplement: Supplementary file 1 [file behavsci-13-00574-s001.zip › Supplementary material S2.pdf]

Supplementary material S2

S2.1 Results for Pleasantness and Want for experience

Behavior as information effect in patients and healthy controls

Pleasantness. Patients perceived the scenarios as less pleasant than healthy controls, diagnostic status ($F_{(1, 188.08)}$ = 124.766, p <0.001). Healthy controls rated scenarios with safety behaviors as less pleasant than scenarios without safety behaviors, safety behavior ($F_{(1, 2843.16)}$ = 90.762, p <0.001). The effect of safety behaviors on pleasantness ratings was smaller in patients than in healthy controls, diagnostic status x safety behavior interaction ($F_{(1, 2843.22)}$ = 22.735, p <0.001).

Want for experience. Patients indicated a lower desire to experience the scenarios than healthy controls, diagnostic status ($F_{(1, 188.01)}$ = 70.345, p <0.001). Healthy controls gave lower Want for experience ratings on scenarios with safety behaviors than on scenarios without safety behaviors, safety behavior ($F_{(1, 2845.03)}$ = 54.65, p <0.001). This behavior as information effect was smaller for patients, diagnostic status x safety behavior interaction ($F_{(1, 2845.10)}$ = 11.839, p <0.001).

Effect of danger information on the behavior as information effect

Pleasantness. Participants found dangerous scenarios less pleasant than safe scenarios, presence of danger ($F_{(1, 2839.14)}$ = 170.4921, p <0.001). The presence of danger also affected the behavior as information effect: The difference in pleasantness ratings between scenarios with and without safety behaviors was larger in safe compared with dangerous scenarios, safety behavior x presence of danger interaction ($F_{(1,2839.19)}$ = 26.6678, p <0.001). Post-hoc comparisons showed that in safe scenarios safety behaviors influenced patients’ and healthy controls’ pleasantness ratings (patients: β = 10.16, SE = 1.71, $t_{(2839)}$ = 5.937, p <0.001, d =

0.11; HC: *β* = 19.11, SE = 1.96, $t_{(2839)}$ = 9.743, *p* <0.001, *d* = 0.18), while in dangerous scenarios safety behaviors only affected the ratings of healthy controls (patients: *p* = 0.7697, *d* = −0.005; HC: *β* = 9.55, SE = 1.96, $t_{(2839)}$ = 4.874, *p* <0.001, *d* = 0.09).

Want for experience. Participants were less eager to experience dangerous scenarios compared with safe scenarios, presence of danger ($F_{(1, 2841.03)}$ = 124.9476, *p* <0.001). The influence of safety behaviors on participants' want to experience the situation was smaller in dangerous compared with safe scenarios, safety behavior x presence of danger interaction ($F_{(1, 2841.03)}$ = 7.1139, *p* = 0.008). Post-hoc comparisons showed that on safe scenarios both patients and healthy controls showed a behaviors as information effect for want to experience ratings (patients: *β* = 9.882, SE = 1.96, $t_{(2841)}$ = 5.054, *p* <0.001, *d* = 0.09 ; HC: *β* = 16.69, SE = 2.24, $t_{(2841)}$ = 7.437, *p* <0.001, *d* = 0.14), while on dangerous scenarios safety behaviors only influenced healthy controls (patients: *p* = 0.8776, *d* = −0.002; HC: *β* = 8.22, SE = 2.24, $t_{(2841)}$ = 3.66, *p* <0.001, *d* = 0.07).

S2.2 Parameter information for the mixed model analyses

Table S1

Model parameters for the LMM with threat ratings as dependent variable and diagnostic status and the presence of safety behaviors as fixed effects.

Predictor	*β*	SE *β*	Standardized *β*	*t*	df	*p*
Diagnostic status (Patients)	40.94	2.95	0.76	13.88	221.58	**<0.001**
Safety behavior (SB)	8.39	1.25	0.18	6.70	2848	**<0.001**
Diagnostic status (Patients) x Safety behavior (SB)	−2.92	1.66	−0.06	−1.76	2848	0.079

Table S2

Model parameters for the LMM with threat ratings as dependent variable and diagnostic status, the presence of safety behaviors and the presence of danger as fixed effects.

Predictor	*β*	SE *β*	Standardized *β*	*t*	df	*p*

Diagnostic status (Patients)	36.96	2.96	0.68	12.48	212.20	**<0.001**
Safety behavior (SB)	11.71	1.59	0.26	7.37	620.35	**<0.001**
Danger (Dangerous)	17.85	1.92	0.39	9.30	398.70	**<0.001**
Diagnostic status (Patients) x Safety behavior (SB)	−1.40	2.11	−0.03	−0.66	620.35	0.508
Safety behavior (SB) x Danger (Dangerous)	−6.66	2.17	−0.13	−3.07	2468.01	**0.002**
Diagnostic status (Patients) x Danger (Dangerous)	7.95	2.55	0.16	3.12	398.70	**0.002**
Diagnostic status (Patients) x Safety behavior (SB) x Danger (Dangerous)	−3.04	2.88	−0.05	−1.06	2468.01	0.290

Table S3

Model parameters for the LMM with pleasantness ratings as dependent variable and diagnostic status and the presence of safety behaviors as fixed effects.

Predictor	*β*	SE *β*	Standardized *β*	*t*	df	*p*
Diagnostic status (Patients)	−28.19	2.32	−0.82	−12.14	281.91	**<0.001**
Safety behavior (SB)	−14.34	1.51	−0.26	−9.53	2843.17	**<0.001**
Diagnostic status (Patients) x Safety behavior (SB)	9.52	2.00	0.15	4.77	2843.23	**<0.001**

Table S4

Model parameters for the LMM with pleasantness ratings as dependent variable and diagnostic status, the presence of safety behaviors and the presence of danger as fixed effects.

Predictor	*β*	SE *β*	Standardized *β*	*t*	df	*p*
Diagnostic status (Patients)	−26.27	2.63	−0.76	−9.97	457.75	**<0.001**
Safety behavior (SB)	−19.11	1.96	−0.34	−9.74	2839.20	**<0.001**
Danger (Dangerous)	−22.88	1.96	−0.41	−11.67	2839.09	**<0.001**
Diagnostic status (Patients) x Safety behavior (SB)	8.94	2.60	0.15	3.44	2839.30	**0.001**
Safety behavior (SB) x Danger (Dangerous)	9.56	2.77	0.15	3.45	2839.14	**0.001**

Diagnostic status (Patients) x Danger (Dangerous)	−3.78	2.60	−0.06	−1.45	2839.16	0.146
Diagnostic status (Patients) x Safety behavior (SB) x Danger (Dangerous)	1.11	3.68	0.01	0.30	2839.19	0.763

Table S5

Model parameters for the LMM with want for experience ratings as dependent variable and diagnostic status and the presence of safety behaviors as fixed effects.

Predictor	*β*	SE *β*	Standardized *β*	*t*	df	*p*
Diagnostic status (Patients)	−22.50	2.49	−0.70	−9.04	293.58	**<0.001**
Safety behavior (SB)	−12.45	1.68	−0.20	−7.39	2845.03	**<0.001**
Diagnostic status (Patients) x Safety behavior (SB)	7.69	2.24	0.11	3.44	2845.11	**0.001**

Table S6

Model parameters for the LMM with want for experience ratings as dependent variable and diagnostic status, the presence of safety behaviors and presence of danger as fixed effects.

Predictor	*β*	SE *β*	Standardized *β*	*t*	df	*p*
Diagnostic status (Patients)	−20.65	2.87	−0.64	−7.18	510.28	**<0.001**
Safety behavior (SB)	−16.69	2.24	−0.27	−7.44	2841.03	**<0.001**
Danger (Dangerous)	−21.97	2.24	−0.36	−9.79	2841.03	**<0.001**
Diagnostic status (Patients) x Safety behavior (SB)	6.80	2.98	−0.05	2.29	2841.03	**0.022**
Safety behavior (SB) x Danger (Dangerous)	8.46	3.17	0.12	2.67	2841.03	**0.008**
Diagnostic status (Patients) x Danger (Dangerous)	−3.70	2.98	−0.05	−1.24	2841.03	0.214
Diagnostic status (Patients) x Safety behavior (SB) x Danger (Dangerous)	1.72	4.21	0.02	0.41	2841.10	0.683

S2.3 Robustness checks

Results when excluding patients with OSFED (n = 38) from the analyses

Table S7

Model parameters for the LMM with threat ratings as dependent variable and with diagnostic status and presence of safety behaviors as fixed effects.

Predictor	β	SE β	Standardized β	*t*	df	*p*
Diagnostic status (Patients)	42.17	3.28	0.77	12.85	176.27	**<0.001**
Safety behavior (SB)	8.39	1.24	0.18	6.75	2278	**<0.001**
Diagnostic status (Patients) x Safety behavior (SB)	−3.15	1.83	−0.06	−1.72	2278	0.085

Table S8

Model parameters for the LMM with threat ratings as dependent variable and with diagnostic status, presence of safety behaviors and presence of danger as fixed effects.

Predictor	β	SE β	Standardized β	*t*	df	*p*
Diagnostic status (Patients)	38.54	3.19	0.70	12.07	170.61	**<0.001**
Safety behavior (SB)	11.71	1.64	0.26	7.13	450.36	**<0.001**
Danger (Dangerous)	17.85	1.90	0.39	9.40	324.27	**<0.001**
Diagnostic status (Patients) x Safety behavior (SB)	−1.97	2.42	−0.04	−0.82	450.36	0.415
Safety behavior (SB) x Danger (Dangerous)	−6.66	2.17	−0.13	−3.07	1974.01	**0.002**
Diagnostic status (Patients) x Danger (Dangerous)	7.26	2.80	0.13	2.59	324.27	**0.010**
Diagnostic status (Patients) x Safety behavior (SB) x Danger (Dangerous)	−2.36	3.19	−0.03	−0.74	1974.01	0.461

Table S9

Model parameters for the LMM with pleasantness ratings as dependent variable and with diagnostic status and presence of safety behaviors as fixed effects.

Predictor	β	SE β	Standardized β	*t*	df	*p*
Diagnostic status (Patients)	−29.40	2.46	−0.88	−11.95	239.41	**<0.001**

Safety behavior (SB)	−14.34	1.53	−0.25	−9.38	2273.17	**<0.001**
Diagnostic status (Patients) x Safety behavior (SB)	10.20	2.25	0.15	4.52	2273.30	**<0.001**

Table S10

Model parameters for the LMM with pleasantness ratings as dependent variable and with diagnostic status, presence of safety behaviors and presence of danger as fixed effects.

Predictor	*β*	SE *β*	Standardized *β*	*t*	df	*p*
Diagnostic status (Patients)	−27.08	2.84	−0.81	−9.55	413.35	**<0.001**
Safety behavior (SB)	−19.11	2.00	−0.34	−9.55	2269.21	**<0.001**
Danger (Dangerous)	−22.88	2.00	−0.41	−11.44	2269.10	**<0.001**
Diagnostic status (Patients) x Safety behavior (SB)	8.99	2.95	0.13	3.04	2269.42	**0.002**
Safety behavior (SB) x Danger (Dangerous)	9.56	2.83	0.15	3.38	2269.15	**0.001**
Diagnostic status (Patients) x Danger (Dangerous)	4.53	2.95	−0.07	−1.53	2269.23	0.125
Diagnostic status (Patients) x Safety behavior (SB) x Danger (Dangerous)	2.36	4.17	0.03	0.56	2269.26	0.572

Table S11

Model parameters for the LMM with want to experience ratings as dependent variable and with diagnostic status and presence of safety behaviors as fixed effects.

Predictor	*β*	SE *β*	Standardized *β*	*t*	df	*p*
Diagnostic status (Patients)	−23.24	2.72	−0.73	−8.54	238.40	**<0.001**
Safety behavior (SB)	−12.45	1.69	−0.20	−7.38	2276	**<0.001**
Diagnostic status (Patients) x Safety behavior (SB)	7.27	2.49	0.10	2.92	2276.08	**0.004**

Table S12

Model parameters for the LMM with want to experience ratings as dependent variable and with diagnostic status, presence of safety behaviors and presence of danger as fixed effects.

Predictor	*β*	SE *β*	Standardized *β*	*t*	df	*p*
Diagnostic status (Patients)	−21.52	3.16	−0.67	−6.80	423.36	**<0.001**

Safety behavior (SB)	−16.69	2.26	−0.27	−7.39	2272	**<0.001**
Danger (Dangerous)	−21.97	2.26	−0.35	−9.74	2272	**<0.001**
Diagnostic status (Patients) x Safety behavior (SB)	6.10	3.32	0.08	1.83	2272	0.067
Safety behavior (SB) x Danger (Dangerous)	8.46	3.19	0.12	2.65	2272	**0.008**
Diagnostic status (Patients) x Danger (Dangerous)	−3.44	3.32	−0.05	−1.04	2272	0.300
Diagnostic status (Patients) x Safety behavior (SB) x Danger (Dangerous)	2.28	4.70	0.02	0.49	2272.07	0.627

Results when excluding healthy controls with an EDE-Q score greater than 2 (n = 29)

Table S13

Model parameters for the LMM with threat ratings as dependent variable and with diagnostic status and presence of safety behaviors as fixed effects.

Predictor	*β*	SE *β*	Standardized *β*	*t*	df	*p*
Diagnostic status (Patients)	49.80	3.03	0.84	16.46	194.18	**<0.001**
Safety behavior (SB)	9.00	1.53	0.20	5.88	2413	**<0.001**
Diagnostic status (Patients) x Safety behavior (SB)	−3.53	1.87	−0.07	−1.89	2413	0.059

Table S14

Model parameters for the LMM with threat ratings as dependent variable and with diagnostic status, presence of safety behaviors and presence of danger as fixed effects.

Predictor	*β*	SE *β*	Standardized *β*	*t*	df	*p*
Diagnostic status (Patients)	43.73	3.14	0.74	13.94	182.72	**<0.001**
Safety behavior (SB)	11.94	1.93	0.26	6.19	560.24	**<0.001**
Danger (Dangerous)	13.66	2.32	0.30	5.89	347.62	**<0.001**
Diagnostic status (Patients) x Safety behavior (SB)	−1.62	2.35	−0.03	−0.69	560.24	0.492
Safety behavior (SB) x Danger (Dangerous)	−5.87	2.66	−0.11	−2.20	2096.65	**0.028**
Diagnostic status (Patients) x Danger (Dangerous)	12.14	2.83	0.25	4.29	347.62	**<0.001**

Diagnostic status (Patients) x Safety behavior (SB) x Danger (Dangerous)	−3.83	3.25	−0.06	−1.18	2096.65	0.239

Table S15

Model parameters for the LMM with pleasantness ratings as dependent variable and with diagnostic status and presence of safety behaviors as fixed effects.

Predictor	*β*	SE *β*	Standardized *β*	*t*	df	*p*
Diagnostic status (Patients)	−31.93	2.64	−0.85	−12.07	235.98	**<0.001**
Safety behavior (SB)	−15.42	1.84	−0.28	−8.38	2408.18	**<0.001**
Diagnostic status (Patients) x Safety behavior (SB)	10.60	2.25	0.18	4.72	2408.21	**<0.001**

Table S16

Model parameters for the LMM with pleasantness ratings as dependent variable and with diagnostic status, presence of safety behaviors and presence of danger as fixed effects.

Predictor	*β*	SE *β*	Standardized *β*	*t*	df	*p*
Diagnostic status (Patients)	−28.37	2.99	−0.76	−9.48	380.41	**<0.001**
Safety behavior (SB)	−20.06	2.40	−0.37	−8.35	2404.23	**<0.001**
Danger (Dangerous)	−19.62	2.40	−0.36	−8.18	2404.08	**<0.001**
Diagnostic status (Patients) x Safety behavior (SB)	9.89	2.93	0.17	3.37	2404.28	**0.001**
Safety behavior (SB) x Danger (Dangerous)	9.29	3.39	0.15	2.74	2404.15	**0.006**
Diagnostic status (Patients) x Danger (Dangerous)	−7.04	2.93	−0.12	−2.40	2404.13	**0.016**
Diagnostic status (Patients) x Safety behavior (SB) x Danger (Dangerous)	1.37	4.15	0.02	0.33	2404.18	0.741

Table S17

Model parameters for the LMM with want for experience ratings as dependent variable and with diagnostic status and presence of safety behaviors as fixed effects.

Predictor	*β*	SE *β*	Standardized *β*	*t*	df	*p*
Diagnostic status (Patients)	−27.02	2.80	−0.77	−9.63	249.95	**<0.001**

Safety behavior (SB)	−14.06	2.08	−0.23	−6.77	2410.03	**<0.001**
Diagnostic status (Patients) x Safety behavior (SB)	9.30	2.54	0.14	3.67	2410.08	**<0.001**

Table S18

Model parameters for the LMM with want for experience ratings as dependent variable and with diagnostic status, presence of safety behaviors and presence of danger as fixed effects.

Predictor	*β*	SE *β*	Standardized *β*	*t*	df	*p*
Diagnostic status (Patients)	−23.49	3.25	−0.67	−7.23	438.45	**<0.001**
Safety behavior (SB)	−17.66	2.77	−0.29	−6.37	2406.03	**<0.001**
Danger (Dangerous)	−18.61	2.77	−0.30	−6.72	2406.03	**<0.001**
Diagnostic status (Patients) x Safety behavior (SB)	7.77	3.38	0.12	2.30	2406.03	**0.022**
Safety behavior (SB) x Danger (Dangerous)	7.19	3.92	0.10	1.83	2406.03	0.067
Diagnostic status (Patients) x Danger (Dangerous)	−7.06	3.38	−0.11	−2.09	2406.03	**0.037**
Diagnostic status (Patients) x Safety behavior (SB) x Danger (Dangerous)	2.99	4.79	0.04	0.63	2406.07	0.532

Results when including anxiety and depression as covariates in the LMM analyses

Table S19

Model parameters for the LMM with threat ratings as dependent variable, diagnostic status and presence of safety behaviors as fixed effects and DASS Anxiety scores as covariate.

Predictor	*β*	SE *β*	Standardized *β*	*t*	df	*p*
Diagnostic status (Patients)	33.02	3.11	0.61	10.61	215.39	**<0.001**
Safety behavior (SB)	8.39	1.25	0.18	6.73	2833	**<0.001**
Diagnostic status (Patients) x Safety behavior (SB)	−2.96	1.66	0.31	−1.79	2833	0.074
Anxiety (covariate)	0.76	0.14	−0.06	5.63	186	**<0.001**

Table S20

Model parameters for the LMM with threat ratings as dependent variable, diagnostic status and presence of safety behaviors as fixed effects and DASS Depression scores as covariate.

Predictor	*β*	SE *β*	Standardized *β*	*t*	df	*p*
Diagnostic status (Patients)	28.80	3.30	0.53	8.73	211.82	**<0.001**
Safety behavior (SB)	8.39	1.25	0.18	6.73	2833	**<0.001**
Diagnostic status (Patients) x Safety behavior (SB)	−2.96	1.66	−0.06	−1.79	2833	0.074
Depression (covariate)	0.78	0.12	0.38	6.51	186	**<0.001**

Table S21

Model parameters for the LMM with threat ratings as dependent variable, diagnostic status, presence of safety behaviors and presence of danger as fixed effects and DASS Anxiety scores as covariate.

Predictor	*β*	SE *β*	Standardized *β*	*t*	df	*p*
Diagnostic status (Patients)	29.42	3.14	0.54	9.36	214.34	**<0.001**
Safety behavior (SB)	11.71	1.59	0.26	7.38	609.57	**<0.001**
Danger (Dangerous)	17.85	1.92	0.39	9.30	392.79	**<0.001**
Diagnostic status (Patients) x Safety behavior (SB)	−1.48	2.11	−0.03	−0.70	609.59	0.482
Safety behavior (SB) x Danger (Dangerous)	−6.66	2.16	−0.13	−3.09	2454.96	**0.002**
Diagnostic status (Patients) x Danger (Dangerous)	7.90	2.55	0.16	3.10	392.79	**0.002**
Diagnostic status (Patients) x Safety behavior (SB) x Danger (Dangerous)	−2.96	2.87	−0.05	−1.03	2454.96	0.302
Anxiety (covariate)	0.73	0.13	0.30	5.50	186	**<0.001**

Table S22

Model parameters for the LMM with threat ratings as dependent variable, diagnostic status, presence of safety behaviors and presence of danger as fixed effects and DASS Depression scores as covariate.

Predictor	*β*	SE *β*	Standardized *β*	*t*	df	*p*
Diagnostic status (Patients)	24.75	3.26	0.46	7.59	213.55	**<0.001**
Safety behavior (SB)	11.71	1.59	0.26	7.38	609.58	**<0.001**

Danger (Dangerous)	17.85	1.92	0.39	9.30	392.78	**<0.001**
Diagnostic status (Patients) x Safety behavior (SB)	−1.48	2.11	−0.03	−0.70	609.58	0.482
Safety behavior (SB) x Danger (Dangerous)	−6.66	2.16	−0.13	−3.09	2454.99	**0.002**
Diagnostic status (Patients) x Danger (Dangerous)	7.90	2.55	0.16	3.10	392.78	**0.002**
Diagnostic status (Patients) x Safety behavior (SB) x Danger (Dangerous)	−2.96	2.87	−0.05	−1.03	2454.99	0.302
Depression (covariate)	0.79	0.12	0.39	6.77	186	**<0.001**

Table S23

Model parameters for the LMM with pleasantness ratings as dependent variable, diagnostic status and presence of safety behaviors as fixed effects and DASS Anxiety scores as covariate.

Predictor	*β*	SE *β*	Standardized *β*	*t*	df	*p*
Diagnostic status (Patients)	−21.95	2.40	−0.64	−9.13	271.13	**<0.001**
Safety behavior (SB)	−14.34	1.50	−0.26	−9.53	2828.13	**<0.001**
Diagnostic status (Patients) x Safety behavior (SB)	9.50	2.00	0.15	4.75	2828.20	**<0.001**
Anxiety (covariate)	−0.60	0.10	−0.39	−6.10	186.27	**<0.001**

Table S24

Model parameters for the LMM with pleasantness ratings as dependent variable, diagnostic status and presence of safety behaviors as fixed effects and DASS Depression scores as covariate.

Predictor	*β*	SE *β*	Standardized *β*	*t*	df	*p*
Diagnostic status (Patients)	−18.27	2.50	−0.53	−7.30	262.60	<0.001
Safety behavior (SB)	−14.34	1.50	−0.26	−9.53	2828.16	<0.001
Diagnostic status (Patients) x Safety behavior (SB)	9.50	2.00	0.15	4.75	2828.24	<0.001
Depression (covariate)	−0.64	0.09	−0.49	−7.40	186.09	<0.001

Table S25

Model parameters for the LMM with pleasantness ratings as dependent variable, diagnostic status, presence of safety behaviors and presence of danger as fixed effects and DASS Anxiety scores as covariate.

Predictor	*β*	SE *β*	Standardized *β*	*t*	df	*p*
Diagnostic status (Patients)	−20.06	2.71	−0.58	−7.41	428.67	**<0.001**
Safety behavior (SB)	−19.10	1.96	−0.34	−9.75	2824.17	**<0.001**
Danger (Dangerous)	−22.88	1.96	−0.41	−11.68	2824.04	**<0.001**
Diagnostic status (Patients) x Safety behavior (SB)	9.01	2.61	0.15	3.46	2824.30	**0.001**
Safety behavior (SB) x Danger (Dangerous)	9.55	2.77	0.15	3.45	2824.11	**0.001**
Diagnostic status (Patients) x Danger (Dangerous)	−3.75	2.60	−0.06	−1.44	2824.13	0.150
Diagnostic status (Patients) x Safety behavior (SB) x Danger (Dangerous)	0.92	3.68	0.01	0.25	2824.17	0.803
Anxiety (covariate)	−0.60	0.10	−0.39	−6.09	186.23	**<0.001**

Table S26

Model parameters for the LMM with pleasantness ratings as dependent variable, diagnostic status, presence of safety behaviors and presence of danger as fixed effects and DASS Depression scores as covariate.

Predictor	*β*	SE *β*	Standardized *β*	*t*	df	*p*
Diagnostic status (Patients)	−16.38	2.80	−0.48	−5.86	402.75	**<0.001**
Safety behavior (SB)	−19.10	1.96	−0.34	−9.75	2824.21	**<0.001**
Danger (Dangerous)	−22.88	1.96	−0.41	−11.68	2824.06	**<0.001**
Diagnostic status (Patients) x Safety behavior (SB)	9.01	2.61	0.15	3.46	2824.34	**0.001**
Safety behavior (SB) x Danger (Dangerous)	9.55	2.77	0.15	3.45	2824.13	**0.001**
Diagnostic status (Patients) x Danger (Dangerous)	−3.75	2.60	−0.06	−1.44	2824.16	0.150
Diagnostic status (Patients) x Safety behavior (SB) x Danger (Dangerous)	0.92	3.68	0.01	0.25	2824.20	0.803

Depression (covariate)	−0.64	0.09	−0.49	−7.39	186.07	**<0.001**

Table S27

Model parameters for the LMM with want for experience ratings as dependent variable, diagnostic status and presence of safety behaviors as fixed effects and DASS Anxiety scores as covariate.

Predictor	*β*	SE *β*	Standardized *β*	*t*	df	*p*
Diagnostic status (Patients)	−19.90	2.77	−0.62	−7.19	264.82	**<0.001**
Safety behavior (SB)	−12.45	1.68	−0.20	−7.40	2830	**<0.001**
Diagnostic status (Patients) x Safety behavior (SB)	7.61	2.24	0.11	3.40	2830.07	**0.001**
Anxiety (covariate)	−0.24	0.11	−0.17	−2.11	186.07	**0.035**

Table S28

Model parameters for the LMM with want for experience ratings as dependent variable, diagnostic status and presence of safety behaviors as fixed effects and DASS Depression scores as covariate.

Predictor	*β*	SE *β*	Standardized *β*	*t*	df	*p*
Diagnostic status (Patients)	−15.67	2.89	−0.49	−5.42	256.54	**<0.001**
Safety behavior (SB)	−12.45	1.68	−0.20	−7.40	2829.97	**<0.001**
Diagnostic status (Patients) x Safety behavior (SB)	7.61	2.24	0.11	3.40	2830.05	**0.001**
Depression (covariate)	−0.43	0.10	−0.35	−4.28	186.02	**<0.001**

Table S29

Model parameters for the LMM with want for experience ratings as dependent variable, diagnostic status, presence of safety behaviors and presence of danger as fixed effects and DASS Anxiety scores as covariate.

Predictor	*β*	SE *β*	Standardized *β*	*t*	df	*p*
Diagnostic status (Patients)	−18.01	3.12	−0.56	−5.77	421.34	**<0.001**
Safety behavior (SB)	−16.69	2.24	−0.27	−7.45	2826	**<0.001**
Danger (Dangerous)	−21.97	2.24	−0.36	−9.81	2826	**<0.001**
Diagnostic status (Patients) x Safety behavior (SB)	6.74	2.98	0.10	2.26	2826	**0.024**

Safety behavior (SB) x Danger (Dangerous)	8.46	3.17	0.12	2.67	2826	**0.008**
Diagnostic status (Patients) x Danger (Dangerous)	−3.76	2.98	−0.06	−1.26	2826	0.206
Diagnostic status (Patients) x Safety behavior (SB) x Danger (Dangerous)	1.69	4.21	0.02	0.40	2826.07	0.687
Anxiety (covariate)	−0.24	0.11	−0.17	−2.12	186.07	**0.034**

Table S30

Model parameters for the LMM with want for experience ratings as dependent variable, diagnostic status, presence of safety behaviors and presence of danger as fixed effects and DASS Depression scores as covariate.

Predictor	*β*	SE *β*	Standardized *β*	*t*	df	*p*
Diagnostic status (Patients)	−13.78	3.23	−0.43	−4.27	394.98	**<0.001**
Safety behavior (SB)	−16.69	2.24	−0.27	−7.45	2825.98	**<0.001**
Danger (Dangerous)	−21.97	2.24	−0.36	−9.81	2825.98	**<0.001**
Diagnostic status (Patients) x Safety behavior (SB)	6.74	2.98	0.10	2.26	2825.98	**0.024**
Safety behavior (SB) x Danger (Dangerous)	8.46	3.17	0.12	2.67	2825.98	**0.008**
Diagnostic status (Patients) x Danger (Dangerous)	−3.76	2.98	−0.06	−1.26	2825.98	0.206
Diagnostic status (Patients) x Safety behavior (SB) x Danger (Dangerous)	1.69	4.21	0.02	0.40	2826.05	0.688
Depression (covariate)	−0.43	0.10	−0.35	−4.29	186.02	**<0.001**
